# Lung vessel connectivity map as anatomical prior knowledge for deep learning-based lung lobe segmentation

**DOI:** 10.1117/1.JMI.11.4.044001

**Published:** 2024-07-09

**Authors:** Simone Bendazzoli, Emelie Bäcklin, Örjan Smedby, Birgitta Janerot-Sjoberg, Bryan Connolly, Chunliang Wang

**Affiliations:** a KTH Royal Institute of Technology, Department of Biomedical Engineering and Health Systems, Huddinge, Sweden; b Karolinska Institutet, Department of Clinical Science, Intervention and Technology, Solna, Sweden; c Karolinska Institutet, Department of Radiology, Solna, Sweden

**Keywords:** pulmonary lobe segmentation, computed tomography, deep learning, 3D segmentation

## Abstract

**Purpose:**

Our study investigates the potential benefits of incorporating prior anatomical knowledge into a deep learning (DL) method designed for the automated segmentation of lung lobes in chest CT scans.

**Approach:**

We introduce an automated DL-based approach that leverages anatomical information from the lung’s vascular system to guide and enhance the segmentation process. This involves utilizing a lung vessel connectivity (LVC) map, which encodes relevant lung vessel anatomical data. Our study explores the performance of three different neural network architectures within the nnU-Net framework: a standalone U-Net, a multitasking U-Net, and a cascade U-Net.

**Results:**

Experimental findings suggest that the inclusion of LVC information in the DL model can lead to improved segmentation accuracy, particularly, in the challenging boundary regions of expiration chest CT volumes. Furthermore, our study demonstrates the potential for LVC to enhance the model’s generalization capabilities. Finally, the method’s robustness is evaluated through the segmentation of lung lobes in 10 cases of COVID-19, demonstrating its applicability in the presence of pulmonary diseases.

**Conclusions:**

Incorporating prior anatomical information, such as LVC, into the DL model shows promise for enhancing segmentation performance, particularly in the boundary regions. However, the extent of this improvement has limitations, prompting further exploration of its practical applicability.

## 1 Introduction

Lung lobe segmentation plays a significant role in the evaluation and treatment of various lung pathologies. This anatomical information becomes crucial for assessing the distribution and extent of pulmonary diseases.^1^^,^^2^ Additionally, this information aids in optimizing treatment planning for such pathologies by focusing on the affected lobes.^3^ Many lung diseases primarily impact specific lung lobes,^4^^,^^5^ underscoring the relevance of lung lobe segmentation in diagnosing and evaluating these conditions.

The lungs consist of five distinct anatomical and functional structures, divided by lobar fissures (left oblique, right oblique, and right horizontal fissures).^6^ The bronchial tree and vascular system are also independent across different lobes.

Lung lobe segmentation entails the identification of fissures between adjacent lobes. This task can be challenging due to factors, such as incomplete fissure delineation,^7^ anatomical variability in fissures,^6^^,^^8^ and overlapping boundaries due to lung diseases.^9^

Given this complexity, several methods in the literature focus on lung fissure detection. For instance, Doel et al.^10^ conducted a comprehensive review of pulmonary lobar segmentation methods, including watershed algorithms,^11^ b-splines,^12^ and surface fitting based on the lobe fissure model.^13^ Ross et al.^14^ introduced a thin-plate spline algorithm for fissure delineation, followed by a maximum *a posteriori* estimation based on an atlas.

These methods have often concentrated on lobe segmentation in healthy subjects, with limited consideration of pathological changes. However, a growing body of literature incorporates anatomical knowledge into the workflow for pathological lungs. For instance, ribs’ curvature^15^ and bronchi/trachea extraction^16^ have been integrated. Anatomical constraints from surrounding organs^17^ and knowledge from the airways and lung vascular system^18^ have also been used.

Deep learning (DL)-based methods have recently gained relevance in medical image processing, including lung lobe segmentation.^19^^–^^21^ These methods aim to improve segmentation performance even in the presence of lung abnormalities. Techniques, such as the P-HNN,^22^ U-Net,^23^ and V-Net,^24^ have been applied to lung lobe segmentation.^25^^–^^28^ These approaches often involve adapting existing architectures and integrating them with innovative strategies to enhance segmentation outcomes in the presence of lung pathologies.

The majority of studies focusing on lobar segmentation have concentrated on chest CT scans obtained during the inspiration breathing phase. This preference is due to the anatomical configuration of the lungs during inspiration: lung volume expands, revealing the overall anatomy and enabling effective examination of the lung region. On the contrary, during expiration, lung volume decreases along with the contraction of lobar fissures. Moreover, due to radiation exposure concerns, expiratory CT scans are generally less analyzed.

This reduced suitability for fissure detection and analysis during expiration has led to limited research and discussion regarding lung lobe segmentation in expiratory chest CT scans. However, recent clinical studies have highlighted the diagnostic superiority of expiration chest CTs for illustrating pathophysiological changes in patients with chronic obstructive pulmonary disease (COPD).^29^ These studies underscore the significance of studying lung anatomical and functional dynamics throughout the entire respiratory cycle, encompassing both inspiration and expiration acquisitions.^30^ Additionally, advancements in lowering CT radiation doses have enabled ethical examination of both inspiration and expiration scans, even in healthy subjects.

Given these factors, this study aims to develop a DL-based method for lung lobar segmentation, focusing specifically on expiration chest CT scans. The goal is to leverage prior anatomical knowledge within the DL model to bridge the segmentation performance gap between inspiration and expiration acquisitions. The main hypothesis is that incorporating prior anatomical knowledge about lung vessels can enhance lobe segmentation. This hypothesis is formulated based on observations of radiologists’ practices when annotating challenging lobes: in cases where the lobar fissure is not distinct, radiologists often visually explore the surrounding region, seeking vascular structures that aid in identifying and delineating boundaries between lobes.

Section 2 introduces the datasets utilized in the study and provides a comprehensive explanation of the proposed approach, presenting various available alternatives. In Sec. 3, the results of the proposed method are presented, focusing on segmentation performance and highlighting the distinctions compared to baseline approaches. In Sec. 4, the main findings are discussed, illustrating the implications and insights gained from the study. Finally, Sec. 5 presents conclusions drawn from the study’s outcomes, along with potential implications and applications of the results.

## 2 Materials and Methods

The aim of this study was to create and validate an automated method for lung lobe segmentation in expiration chest CT scans. The primary goal was to investigate whether the segmentation accuracy in expiration CT scans could be enhanced by incorporating prior anatomical information into the DL model. This anatomical information was represented as a vector field within the lung’s vascular region, capturing vessel connectivity. This representation was based on the assumption that lung vessels in different lobes have distinct orientations, a premise supported by clinical practice where radiologists often rely on vascular patterns to identify lobar boundaries in challenging cases.

In our study, we introduced the lung vessel connectivity (LVC) map as an additional input channel to the model to assess whether it could enhance the segmentation performance. We employed three distinct network configurations, all based on the U-Net architecture. To comprehensively evaluate the impact of prior anatomical knowledge and the breathing phase, we conducted an ablation study. This study involved treating inspiration and expiration CT volumes separately, enabling a comparison of the same network architecture’s performance under different breathing phases.

Furthermore, we tested the robustness of our proposed approach on two distinct datasets: SCAPIS and COVID-19. These datasets encompass a range of conditions, including lung lesions, pulmonary diseases such as COPD, and emphysema. This diverse set of data allowed us to investigate how our segmentation approach performed in the presence of these various factors.

### 2.1 Datasets

The study utilized two distinct datasets, each serving specific purposes.

1. *Swedish CArdioPulmonary bioImage Study (SCAPIS)*. This dataset comprises a subset of 59 subjects selected from the SCAPIS study within the Stockholm Region. For each subject, two chest CT acquisitions are available, representing both the inspiration and expiration breathing phases. This dataset includes a total of 118 CT scans, facilitating a performance comparison between the two breathing phases. It covers both healthy subjects (46) and individuals with pathological lung conditions (13), with diagnoses including chronic airflow limitation (11 subjects), emphysema (5 subjects), and COPD (3 subjects).
2. *COVID-19*.^31^ This dataset includes 10 subjects with varying degrees of COVID-19-related lesions in the lung region. It was employed to evaluate the performance of the proposed methods when dealing with ground-glass opacities and mixed consolidation lesions commonly associated with COVID-19.

#### 2.1.1 Data annotation

To generate ground truth data for the DL model, both the SCAPIS and COVID-19 datasets underwent semiautomatic annotation processes. An experienced radiologist supported this annotation task using an interactive tool available in MiaLab, a medical imaging software developed in-house.

The annotation tool for lung lobe segmentation relies on the thin plate spline (TPS) algorithm. Initially, the lung region is automatically segmented through a series of steps involving threshold-based and model-based level set segmentation, following the approach outlined in Ref. 32. In the final stage of the workflow, the user manually annotates the fissure boundaries by placing landmarks along the lobar fissures. Subsequently, a TPS function is computed based on these fissure annotations, enabling the interpolation and deformation of a 3D plane to align with the manually annotated fissure boundaries.

### 2.2 Lung Vessel Connectivity

Several studies have explored the concept of anatomic lobe independence to facilitate the lung lobe segmentation task, as mentioned in Refs. 11 and 18. This independence arises from the fact that independent bronchial branches and vessel subsystems belong to different lobes, and they can be leveraged to guide and assist fissure detection and lobe segmentation.

In this study, the lung vascular system is incorporated into the segmentation task as prior anatomical information, which the network can utilize to delineate the lobes effectively. This approach involves two key steps. First, a fuzzy connectedness (FC) map is generated from the chest CT volume. Subsequently, this map is further processed to derive the LVC map.

#### 2.2.1 Fuzzy connectedness map

In this method, the FC map for the lung vessels is generated using an algorithm introduced in Ref. 33, originally designed for coronary artery segmentation. The algorithm is briefly described as follows.

1. A seed point is manually placed inside the heart region to initiate the FC algorithm.
2. A threshold window of 0 to 200 hounsfield units (HU) is applied to restrict the algorithm’s propagation exclusively within the vascular region voxels. Any voxels outside this window are marked as “deactivated” and are not included in the algorithm.
3. The algorithm’s propagation employs a growing approach. It starts from the seed point and examines the 27-neighborhood voxels. Iteratively, a cost function is optimized to identify the strongest connectivity for each voxel. This process is repeated for all 27 neighboring voxels.
4. The algorithm continues until all the “active” voxels have been analyzed, and their respective cost functions have been optimized.
5. The voxel values in the FC map fall within the range of 0 to 26, indicating the neighbor with the strongest connectivity. Each value corresponds to a specific neighbor, represented as a 3D offset from the considered voxel (refer to Table 1 for details).

**Fig. 1:**
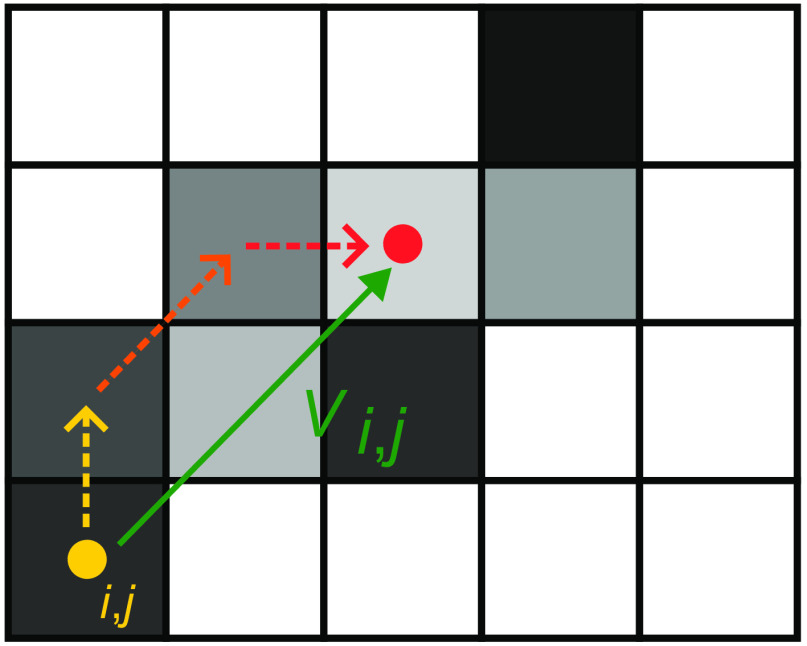
Fuzzy connectivity map example. Only voxels within 0 to 200 HU are considered and optimized. 3-GSN algorithm, highlighting the three iterative steps.

This FC map serves as a representation of LVC, aiding in the subsequent lung lobe segmentation task (Fig. 1).

$${\mathrm{fc}}_{\text{map}}(n)=\{\begin{array}{ll} 0-12,14-26, & \text{if}\;0<\mathrm{CT}[n]<200\\ 13, & \text{otherwise} \end{array}.$$ (1)

**Table 1:** Neighborhood values and corresponding 3D offsets.

Index	3D offset (±x,±y,±z)	Index	3D offset	Index	3D offset
0	$-1,-\overset{\to }{1},-1$	9	$0,-\overset{\to }{1},-1$	18	$1,-\overset{\to }{1},-1$
1	$-1,-\overset{\to }{1},0$	10	$0,-\overset{\to }{1},0$	19	$1,-\overset{\to }{1},0$
2	$-1,-\overset{\to }{1},1$	11	$0,-\overset{\to }{1},1$	20	$1,-\overset{\to }{1},1$
3	$-1,\overset{\to }{0},-1$	12	$0,\;\overset{\to }{0},1$	21	$1,\overset{\to }{0},-1$
4	$-1,\overset{\to }{0},0$	13	$0,\;\overset{\to }{0}0$	22	$1,\overset{\to }{0},0$
5	$-1,\overset{\to }{0},1$	14	$0,\;\overset{\to }{0},1$	23	$1,\;\overset{\to }{0},1$
6	$-1,\overset{\to }{1},-1$	15	$0,\overset{\to }{1},-1$	24	$1,\overset{\to }{1},-1$
7	$-1,\overset{\to }{1},0$	16	$0,\overset{\to }{1},0$	25	$1,\overset{\to }{1},0$
8	$-1,\overset{\to }{1},1$	17	$0,\overset{\to }{1},1$	26	$1,\overset{\to }{1},1$

#### 2.2.2 Lung vessel connectivity map

The LVC map is generated from the FC map and serves to indicate the direction of the strongest connectivity for each voxel within the lung vessel region.

Due to the discrete nature of 3D images, a discretization error arises when assessing the voxel’s strongest connectivity. This error occurs because there are only 26 neighbors for each voxel, resulting in a limited range of values for connectivity directions.

To address this discretization error, we introduce the concept of the “X’th generation strongest neighborhood” (x-GSN):

Definition 2.1 (x-GSN). The x-GSN of a voxel (x,y,z) is defined as the voxel that can be reached through x iterative steps, where at each step, the direction of the strongest connectivity is followed.

Taking the x-GSN into account when assessing the strongest neighborhood connectivity significantly reduces the discretization error. This is because it expands the number of possible directions to $26^{x}$. In this particular approach, we chose to use the third generation strongest neighborhood (3-GSN), denoted as x=3.

To create the LVC map, we first locate the 3-GSN of each voxel. Next, we calculate the distance vector represented as $V_{x,y,z}$ between the two voxels. Finally, the LVC vector, which indicates the direction of the strongest connectivity, is generated by normalizing the vector $V_{x,y,z}$.

The resulting LVC map is a three-channel 3D volume, with each voxel containing the normalized vector components as follows:

$${\overset{\to }{V}}_{i,j,k}=[\begin{array}{c} v_{x}\\ v_{y}\\ v_{z} \end{array}]\;\forall \;V_{i,j,k}\in {\mathrm{LVC}}_{\left(3,H,W,D\right)}.$$

The vectors are oriented from the center of each voxel toward the direction of the strongest connectivity within the neighborhood, with only the segmented vessel voxels being considered.

The presented algorithms [Algorithm 1 and Eq. (1)] have two main implications in the LVC map.

**Fig. 2:**
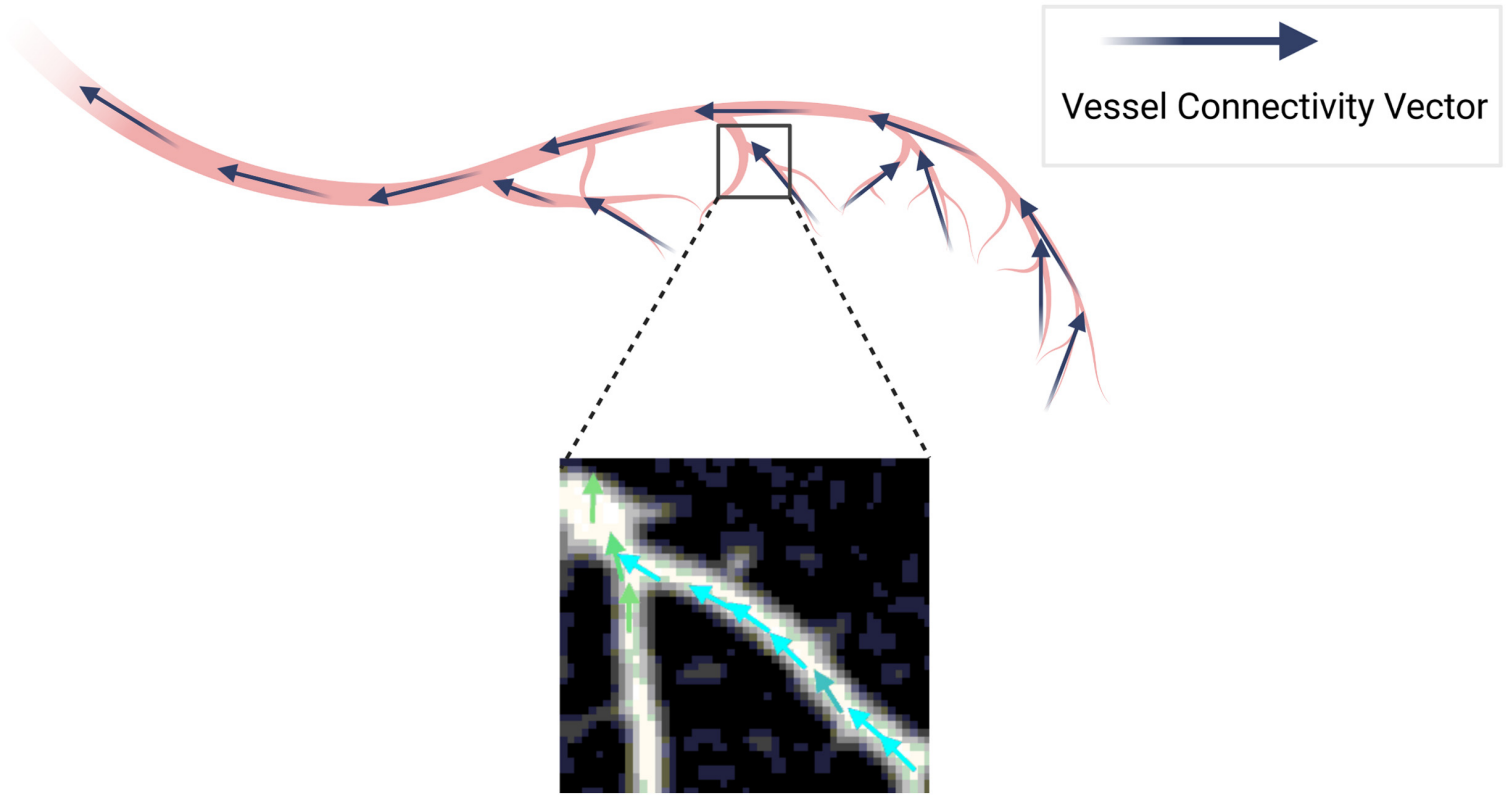
LVC vector field: vector orientation in the vascular tree.

1. Vectors outside the lung vessel segmented regions are set to [0,0,0] (direction 13 in the FC map indicates the direction toward the same voxel).
2. Since the seed point is inserted at the heart apex, the connectivity vectors point, on average, from distal vessel capillaries toward the heart (Fig. 2).

**Algorithm 1:** 3-GSN algorithm to generate the LVC vector

*generations* ← 3
$V_{i}\leftarrow (x,y,z)$
$V_{f}\leftarrow (x,y,z)$
**for**gen←1 to *generations* **do**
$V_{f}\leftarrow argmax_{V_{f}}\;fc(V_{f})$
**end for**
${\overset{\to }{V}}_{x,y,z}=\frac{\left(V_{f}-V_{i}\right)}{\left\|(V_{f}-V_{i})\right\|}$
where $fc(V_{f})$ returns the neighbor voxel with the strongest connectivity, computed following Eq. (1).

In relation to the inclusion of the LVC map in the proposed method, we hypothesize that the LVC vectors can assist the DL network in distinguishing and separating different lung lobes. To provide evidence for this hypothesis, we analyzed the average vector orientation distribution across the different lobes, aiming to demonstrate that LVC vectors belonging to different lobes have well-defined and separated average orientations. The average vector orientation ${\vec{V}}_{{\mathrm{avg}}_{j}}$ for each vascular lobe subsystem j is represented in the following equation:

$$\overset{\to }{V}{\text{sum}}_{j}=\overset{n}{\underset{i=1}{\sum }}[\begin{array}{c} {\overset{\to }{v}}_{i,x}\\ {\overset{\to }{v}}_{i,y}\\ {\overset{\to }{v}}_{i,z} \end{array}],\;{\overset{\to }{v}}_{i}\in j,\;j\in [\mathrm{LU},\mathrm{LL},\mathrm{RU},\mathrm{RM},\mathrm{RL}],\;\overset{\to }{V}{\mathrm{avg}}_{j}=\frac{\overset{\to }{V}{\text{sum}}_{j}}{\left\|\overset{\to }{V}{\text{sum}}_{j}\right\|}.$$ (2)

The computational cost for generating the LVC map was ∼2  min per subject on average, on an AMD Ryzen 9 12-core processor.

### 2.3 Deep Learning Framework

In this study, we propose a DL method to improve the lung lobe segmentation performance by including an LVC map as an additional input information. In order to test this hypothesis, we investigated several deep-learning approaches for the lung lobe segmentation task. The methods are all based on the U-Net architecture. More specifically, they all share the common nnU-Net framework.^34^ This is to unify the experiments in relation to a common framework for the preprocessing steps, network architecture, and hyperparameter tuning.

#### 2.3.1 nnU-Net specifications

Following the method presented in Ref. 34, the image resolution distribution within the dataset is analyzed, and the image volumes are resampled according to the median resolution. Following the image resampling, an image normalization step is performed; for the CT modality, the intensity voxel distribution in the dataset is analyzed, and the voxel values are clipped within the [0.5, 99.5] percentile range followed by a z-score normalization:

$$I_{x,y,z}=\{\begin{array}{ll} I_{0.5p}, & \text{if}\;I_{x,y,z}<I_{0.5p}\\ I_{99.5p}, & \text{if}\;I_{x,y,z}>I_{99.5p}\\ I_{x,y,z}, & \text{otherwise} \end{array},$$ (3)

$$I_{\text{norm}}=\frac{I_{x,y,z}-\mu }{\sigma }.$$ (4)

For the LVC maps, no intensity normalization is performed since the three-dimensional vector components are already normalized within the range [−1,1].

As described in Ref. 34, data augmentation is performed at training time: the applied techniques are random rotations, random scaling, random elastic deformations, and gamma correction augmentation. In Table 2, the other training hyperparameter configurations are specified.

**Table 2:** Training configuration.

Hyperparameter	Value
Optimizer	Adam (default)
Learning rate	0.01
Loss function	Combined Dice-cross entropy (default)
Epochs	1000 (default)
Iterations/epoch	250 (default)
Batch size	2

#### 2.3.2 Network architectures

**Fig. 3:**
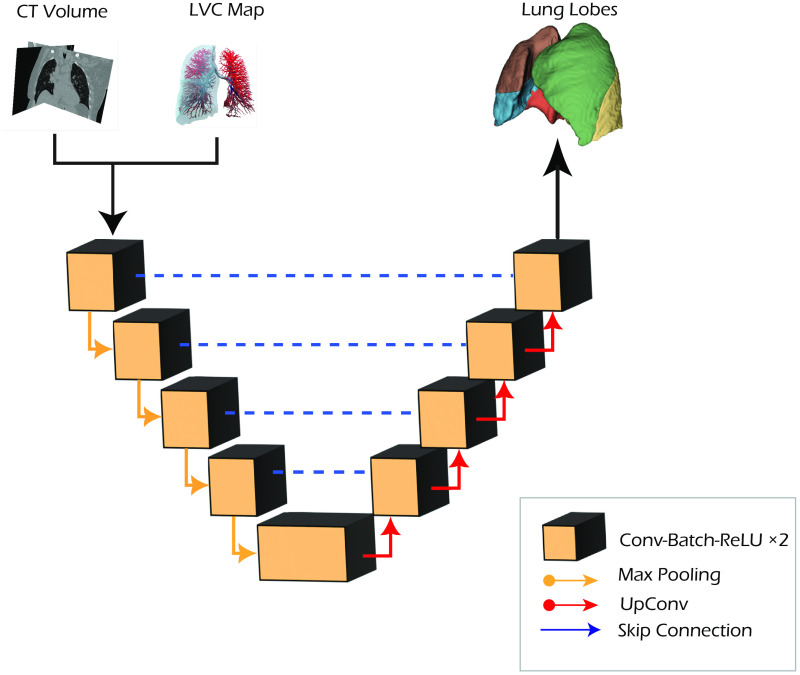
U-Net network architecture.

To evaluate the LVC contribution with respect to different network configurations and inputs, we performed four separated set of experiments: baseline, LVC inclusion, multitasking, and cascade (Fig. 3).

In the baseline approach, the standard nnU-Net pipeline is adopted and trained for a single channel input CT volume, predicting the five lung lobes. In the second configuration (LVC), the LVC map is included as an additional input channel in the network (four input channels: one for the CT volume + three for the LVC map).

**Fig. 4:**
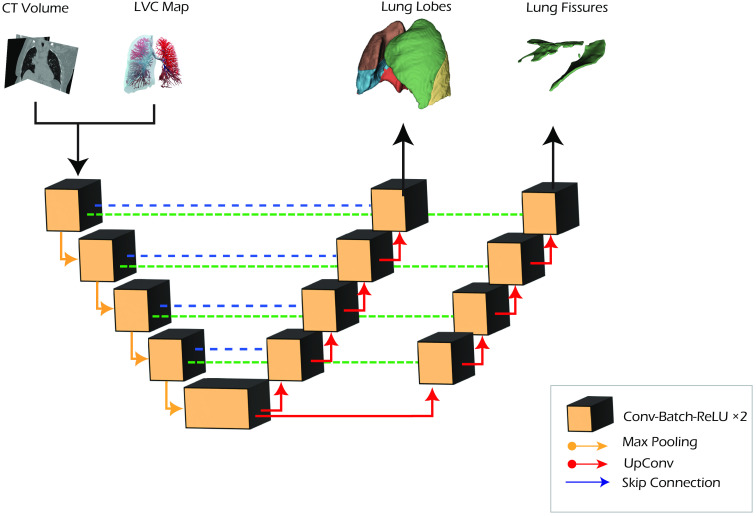
Multitasking U-Net network architecture.

In the multitasking approach, a second decoding branch is added to the original U-Net architecture to predict the lung lobes and the fissure segmentation (Fig. 4). This architecture aims to guide the network in learning common anatomical features in the contracting path. In contrast, the network focuses on learning separated contextual features for the two segmentation tasks (lobe and fissure segmentation) in the expanding paths. The global loss is computed as a weighted sum of the two single task losses:

$$L_{\text{global}}=0.7*L_{\text{lobe}}+0.3*L_{\text{fissure}}.$$

The loss weights for the two terms were empirically assigned by considering the importance of the two tasks. To ensure the justification and robustness of the chosen loss weights, three different combinations were selected and compared to identify the optimal configuration: (0.5, 0.5), (0.7, 0.3), and (0.9, 0.1).

**Fig. 5:**
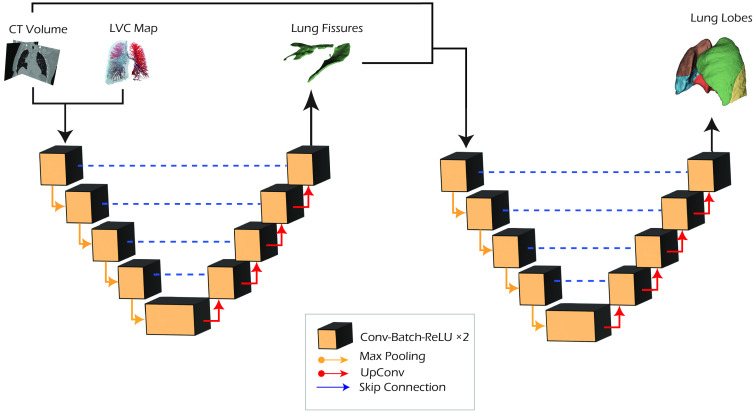
Cascade U-Net network architecture.

Finally, in the cascade configuration, a two-step approach is adopted: in the first step, the CT volume is used, alongside the LVC map, to train a model used for the fissure segmentation task. The CT volume and the predicted fissure mask are subsequently provided as input to a second U-Net, trained for the lobe segmentation task (Fig. 5). For this architecture, the assumption is that the learning process can benefit from splitting the task into subsequent steps: in the first step, the anatomical information (from CT and LVC volumes) is processed by a network to segment the lobar fissures, whereas a second network focuses on the lobe segmentation task, assuming prior knowledge about the fissure location. The two models are independently trained, as they are combined at test time.

The fivefold cross-validation process required an average of 110 h per fold for each model, utilizing a NVIDIA RTX 3090 GPU, resulting in a total of 550 h per experiment. Since two models are generated for the Cascade experiments, the total time required is doubled to 1100 h.

## 3 Results

In order to investigate possible performance differences in relation to the breathing phases, two different models are separately trained for the two breathing phases (inspiration and expiration, 59 volumes each). In contrast, only the expiration cases are considered for the multitasking and the cascade experiments since in this case the aim is to evaluate the performance of different model configurations in respect to expiration cases. For each of the experiments described above, fivefold cross validation is performed on 48 cases, whereas 11 cases are used to evaluate the different model performances on a test set of unseen cases.

To better validate the different lung lobe model performances in relation to the breathing phases, the trained models are also evaluated on a cross-test set: the models trained on expiration cases are tested on the corresponding 11 unseen inspiration test cases and vice versa.

Finally, a model validation on the COVID-19 dataset is performed. Here the specific aim is to evaluate lung lobe segmentation performances in the presence of COVID-19 lesions.

**Fig. 6:**
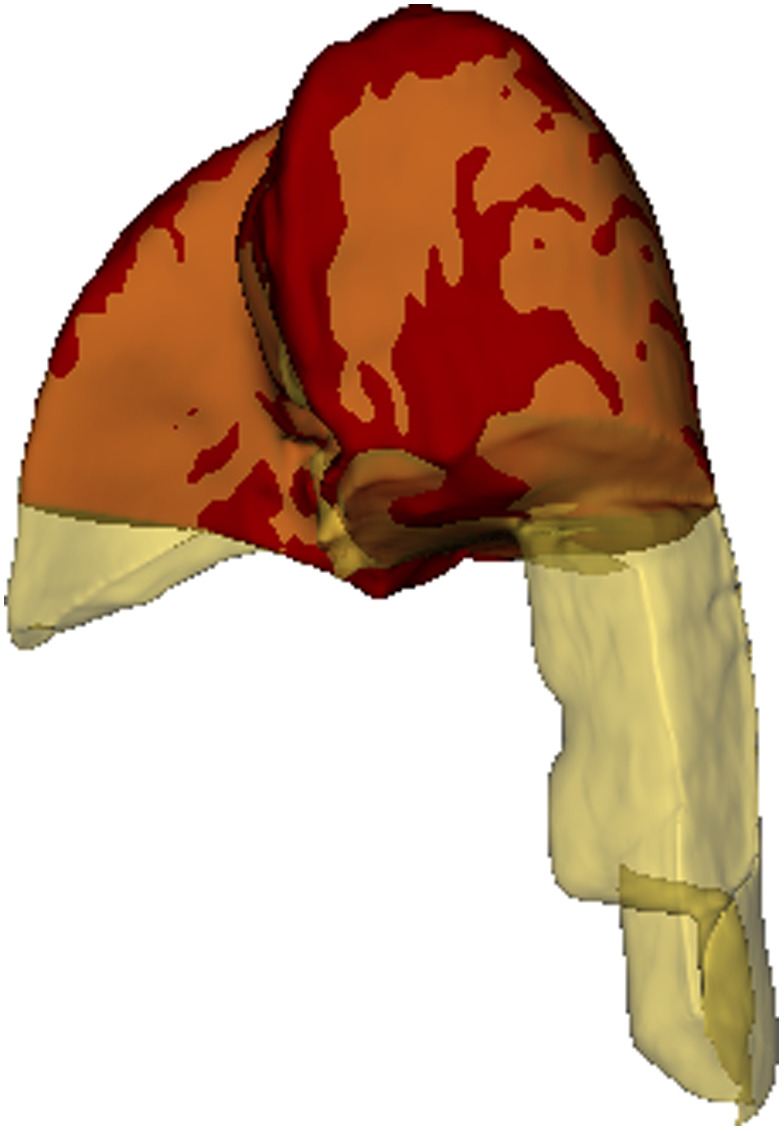
Volumetric overlap between the right-upper annotated lobe (in red) and the predicted lobe (in yellow): despite a Dice score of 0.85, the prediction visual appearance suggests a poor segmentation performance, correctly reported by an ASD of 22 mm.

To evaluate the segmentation performance of all the models presented above, the predicted lobe segmentation masks are compared with the corresponding manually annotated masks. We selected the Dice score and average surface distance (ASD) as the two metrics to represent the lung lobe segmentation performances: Dice provides an indication of the volumetric overlap between the two segmentation masks, whereas ASD provides a better evaluation of the segmentation results in terms of distance between the boundary regions. The size of the segmented regions can explain this choice: large volumes, such as the lung lobes, can easily reach Dice values above 0.8 since large portions of the volumes overlap. Consequently, a more accurate evaluation of large segmented regions can be achieved by focusing on the boundary regions, measuring and reporting the distance between the two surfaces. As anticipated in Sec. 2.3.2, the method and results evaluation are performed in three distinct approaches (Fig. 6).

First, in a single-phase approach, the models are separately trained and compared between the two breathing phases. Accordingly, the LVC contribution for the two breathing phases is evaluated. In addition, the segmentation results from the different model architectures (single U-Net, multitasking U-Net, and cascade U-Net) are compared.

Second, in the interphase approach, we investigated the model generalization capabilities in relation to the breathing phase. More specifically, the models trained with CT volumes from only one of the two breathing phases were tested on CT volumes from the other phase. As in a single-phase approach, we analyzed the LVC contribution in improving the model generalization ability, alongside the impact of the different model architectures.

Finally, we tested the trained models on 10 COVID-19 cases, evaluating the segmentation predictions and comparing them in relation to the breathing phases and the LVC contribution.

### 3.1 LVC Evaluation

Together with the model evaluation, we decided to evaluate also the correctness of our main hypothesis, i.e., LVC vectors exhibit different average orientations for different lobes. In order to evaluate this assumption, we performed a multivariate analysis of the variance (MANOVA) on the spherical coordinates (azimuth and elevation) of the LVC average vectors in relation to the different lung lobes.

As presented in Table 3, this statistical analysis confirms our hypothesis, strongly suggesting that the LVC orientation can provide useful prior information for discriminating the lung lobes.

**Table 3:** MANOVA analysis, performed on SCAPIS cases during expiration and inspiration phases to explore the influence of lung lobe locations on the spherical coordinates of the LVC map. This examination aids in understanding the variability of LVC information across different lung lobes.

Dataset	Effect	Statistic	Value	Pr>F
SCAPIS-In	Lung lobe	Wilks’ lambda	0.879	$6.89\times 10^{-9}$
SCAPIS-Ex	Lung lobe	Wilks’ lambda	0.910	0.000001

### 3.2 Single-Phase Evaluation

Table 4 compares the average Dice scores of a single-phase evaluation, indicating no significant difference between the inspiration and the expiration models. Furthermore, the segmentation performance appeared unaffected by the LVC inclusion and the different architecture choices.

**Table 4:** Single-phase comparison of mean Dice scores, by lobe and globally (in bold).

	LU	LL	RU	RM	RL	Global
Baseline-IN	0.976	0.970	0.965	0.936	0.972	**0.964**
Baseline-EX	0.965	0.963	0.960	0.932	0.968	**0.958**
LVC-IN	0.977	0.973	0.967	0.935	0.974	**0.965**
LVC-EX	0.969	0.966	0.964	0.935	0.971	**0.961**
Cascade-EX	0.964	0.964	0.962	0.936	0.970	**0.959**
MT-EX	0.969	0.967	0.965	0.937	0.971	**0.962**

Abbreviations: IN, inspiration; EX, expiration; and MT, multitasking.

**Fig. 7:**
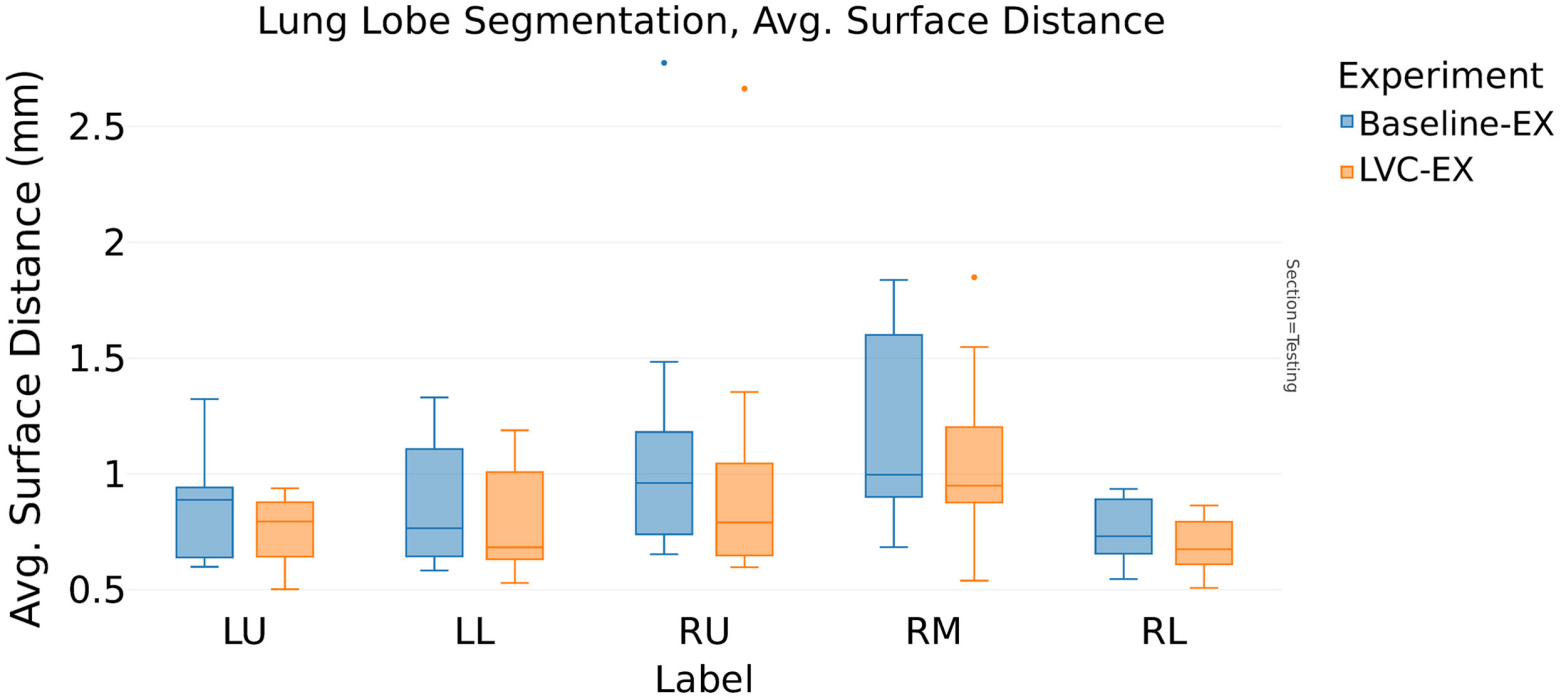
Comparison between baseline U-Net (baseline-EX) and LVC U-Net (LVC-EX) in expiration phase: ASD (mm).

**Fig. 8:**
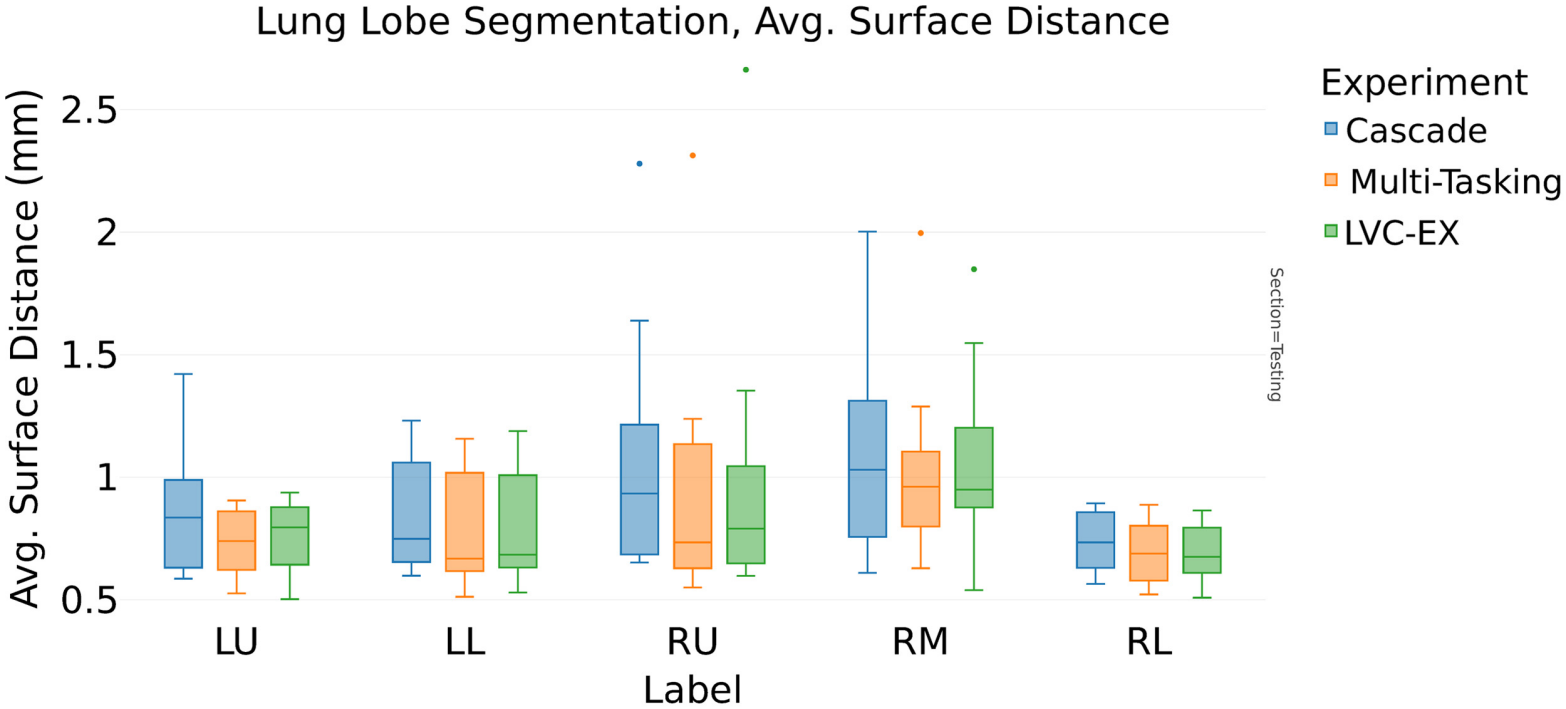
LVC U-Net, cascade, and multitasking comparison in expiration phase: ASD (mm).

In contrast, the analysis of Table 5 (ASD) reveals interesting distinctions. For the expiration model, the LVC inclusion reduces the ASD from 0.95 to 0.86 mm (−9.5%, Fig. 7), whereas in the inspiration model, the LVC positively affects the ASD score reducing it by 7% (from 1.07 to 1.0 mm). The multitasking architecture choice further reduces the ASD to 0.84 mm (−12% from the baseline model, Fig. 8).

**Table 5:** Single-phase comparison of mean ASD (mm), by lobe and globally (in bold).

Metric	LU	LL	RU	RM	RL	Global
Baseline-IN	0.862	0.892	1.451	1.274	0.891	**1.074**
Baseline-EX	0.855	0.850	1.120	1.200	0.746	**0.954**
LVC-IN	0.791	0.806	1.339	1.204	0.844	**0.997**
LVC-EX	0.750	0.776	1.016	1.063	0.695	**0.860**
Cascade-EX	0.870	0.832	1.066	1.109	0.733	**0.922**
MT-EX	0.733	0.776	0.964	1.031	0.691	**0.839**

Abbreviations: IN, inspiration; EX, expiration; and MT, multitasking.

### 3.3 Interphase Validation

**Fig. 9:**
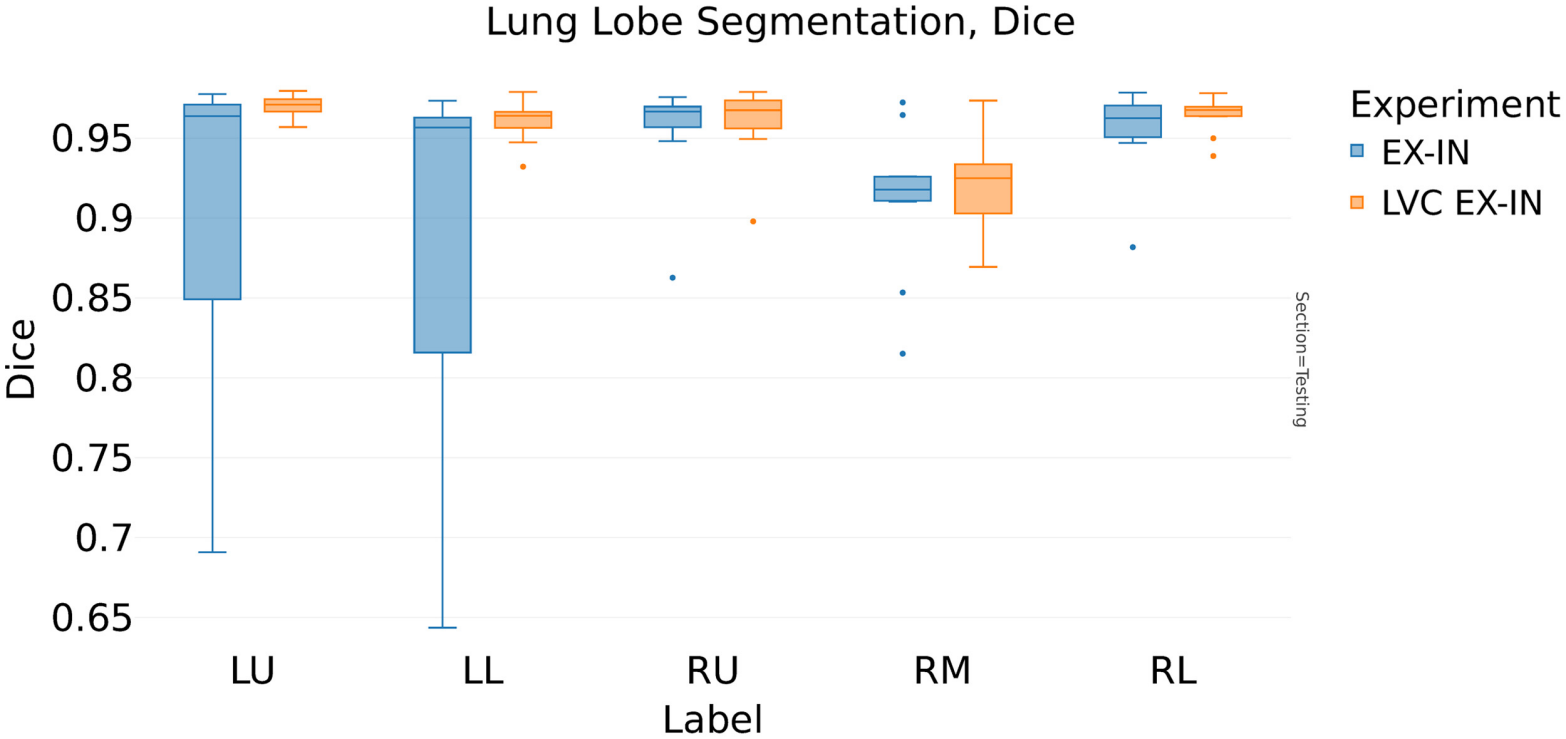
Interphase validation Dice score: comparison between baseline U-Net and LVC U-Net (LVC EX-IN), trained in expiration phase and validated in inspiration phase (EX-IN). Note the underperformance of the baseline model (EX-IN, in blue) compared to the LVC model (LVC EX-IN, in orange) on the LU and LL lobes.

In this second approach, we investigated the ability of the models to generalize successfully with respect to the other breathing phases. As represented in Table 6, LVC has a positive impact on this ability of the expiration model (the average Dice score increases from 0.92 to 0.95, Fig. 9). On the contrary, no significant improvement in the generalization capability can be observed when including LVC information in the inspiration model.

**Table 6:** Interphase comparison of mean Dice scores, categorized by lobe and globally (in bold).

Metric	LU	LL	RU	RM	RL	Global
IN-EX	0.960	0.955	0.943	0.927	0.961	**0.949**
LVC IN-EX	0.962	0.957	0.953	0.922	0.965	**0.952**
EX-IN	0.907	0.887	0.955	0.912	0.955	**0.923**
LVC EX-IN	0.970	0.960	0.961	0.923	0.964	**0.955**
Cascade EX-IN	0.920	0.897	0.958	0.907	0.954	**0.927**
MT EX-IN	0.970	0.962	0.963	0.931	0.967	**0.959**

Abbreviations: IN-EX, model trained in inspiration and tested in expiration; EX-IN, model trained in expiration and tested in inspiration; and MT, multitasking.

**Fig. 10:**
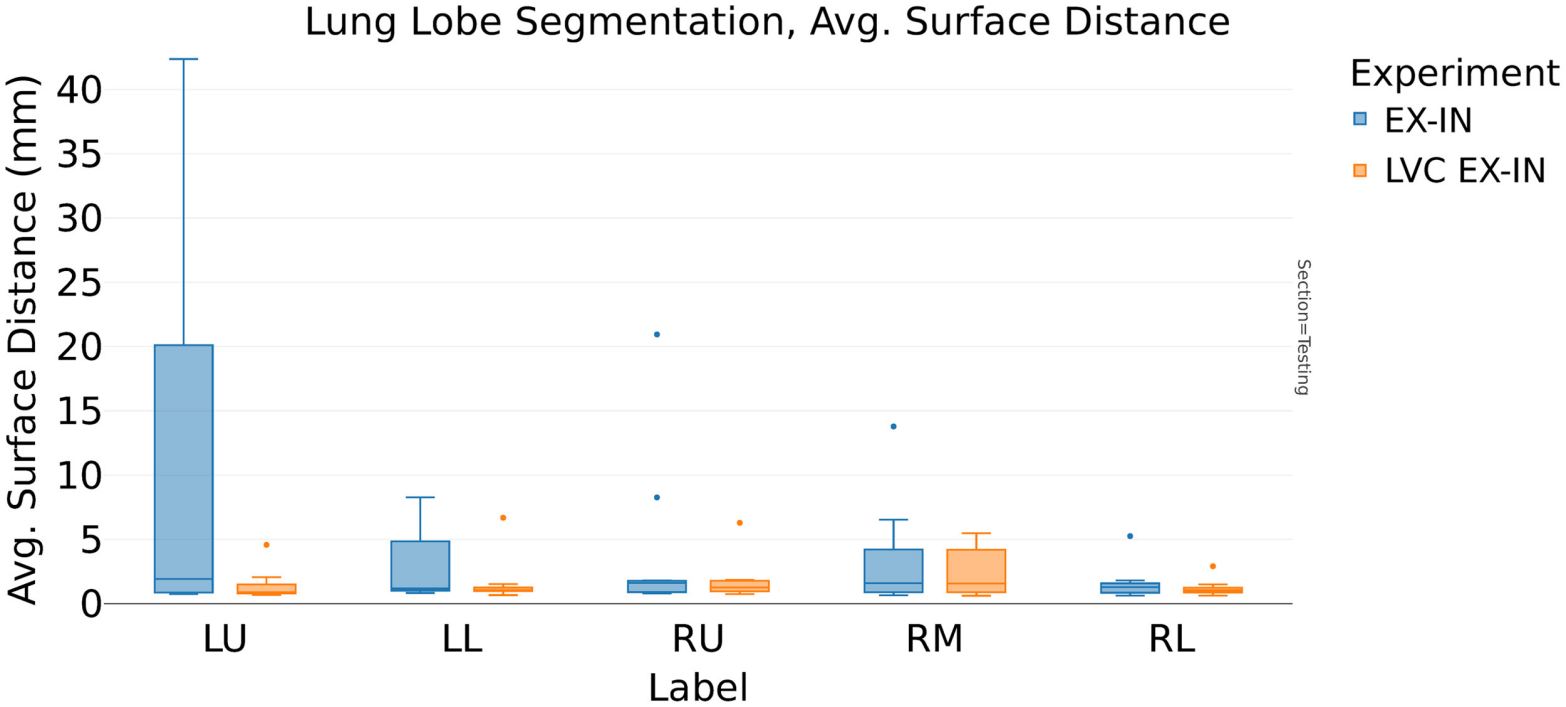
Interphase validation ASD: comparison between baseline U-Net and LVC U-Net (LVC EX-IN), trained in expiration phase and validated in inspiration phase (EX-IN). Similar to the observations in the Dice score figure, note the underperformance of the baseline model (EX-IN, in blue) compared to the LVC model (LVC EX-IN, in orange) on the LU and LL lobes.

**Fig. 11:**
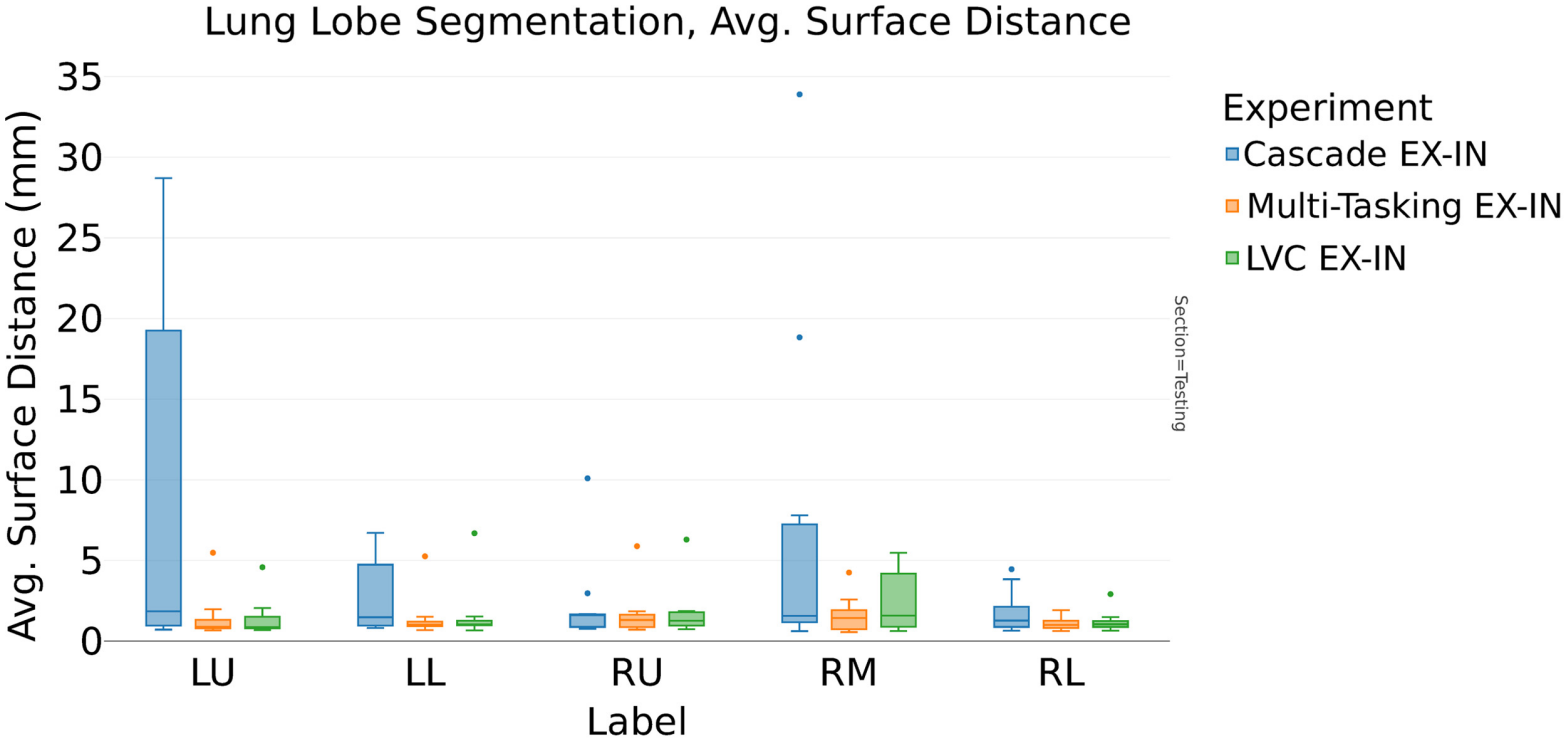
Interphase validation ASD: LVC U-Net (LVC EX-IN), cascade, and multitasking model comparison trained in expiration phase and validated in inspiration phase.

In addition, Table 7 provides further evidence for the LVC ability to provide better generalization performances; for the expiration model, the ASD is reduced from 4.3 to 2.1 mm (Fig. 10). Furthermore, we achieved an additional ASD performance improvement by choosing the multitasking architecture (from 4.3 to 1.4 mm, Fig. 11).

**Table 7:** Interphase comparison of mean ASD (mm), categorized by lobe and globally (in bold).

Metric	LU	LL	RU	RM	RL	Global
IN-EX	0.974	1.037	1.635	1.305	0.941	**1.178**
LVC IN-EX	0.904	1.004	1.276	1.454	0.842	**1.096**
EX-IN	9.999	2.911	3.685	3.219	1.561	**4.275**
LVC EX-IN	1.357	1.577	1.712	2.359	1.211	**2.054**
Cascade EX-IN	9.304	2.592	2.185	6.776	1.796	**4.531**
MT EX-IN	1.424	1.423	1.620	1.572	1.070	**1.422**

Abbreviations: IN-EX, model trained in inspiration and tested in expiration; EX-IN, model trained in expiration and tested in inspiration; and MT, multitasking.

### 3.4 COVID-19 Validation

The LVC effect on the model segmentation performances in COVID-19 cases is analyzed in Tables 8 and 9.

**Table 8:** COVID-19 comparison of mean Dice scores, categorized by lobe and globally (in bold).

Metric	LU	LL	RU	RM	RL	Global
IN-COVID19	0.958	0.940	0.918	0.856	0.940	**0.922**
EX-COVID19	0.943	0.927	0.916	0.881	0.938	**0.921**
LVC IN-COVID19	0.959	0.950	0.919	0.842	0.935	**0.921**
LVC EX-COVID19	0.958	0.950	0.922	0.880	0.952	**0.932**

Abbreviations: IN-COVID19, model trained in inspiration and tested in COVID-19 and EX-COVID19, model trained in expiration and tested in COVID.

**Table 9:** COVID-19 comparison of mean ASD (mm), categorized by lobe and globally (in bold).

Metric	LU	LL	RU	RM	RL	Global
IN-COVID19	1.249	1.495	4.213	2.005	1.479	**2.089**
EX-COVID19	4.318	1.979	5.489	2.047	1.554	**3.077**
LVC IN-COVID19	1.072	1.353	3.801	4.496	1.652	**2.475**
LVC EX-COVID19	1.510	1.533	4.014	2.073	1.364	**2.099**

Abbreviations: IN-COVID19, model trained in inspiration and tested in COVID-19 and EX-COVID19, model trained in expiration and tested in COVID.

**Fig. 12:**
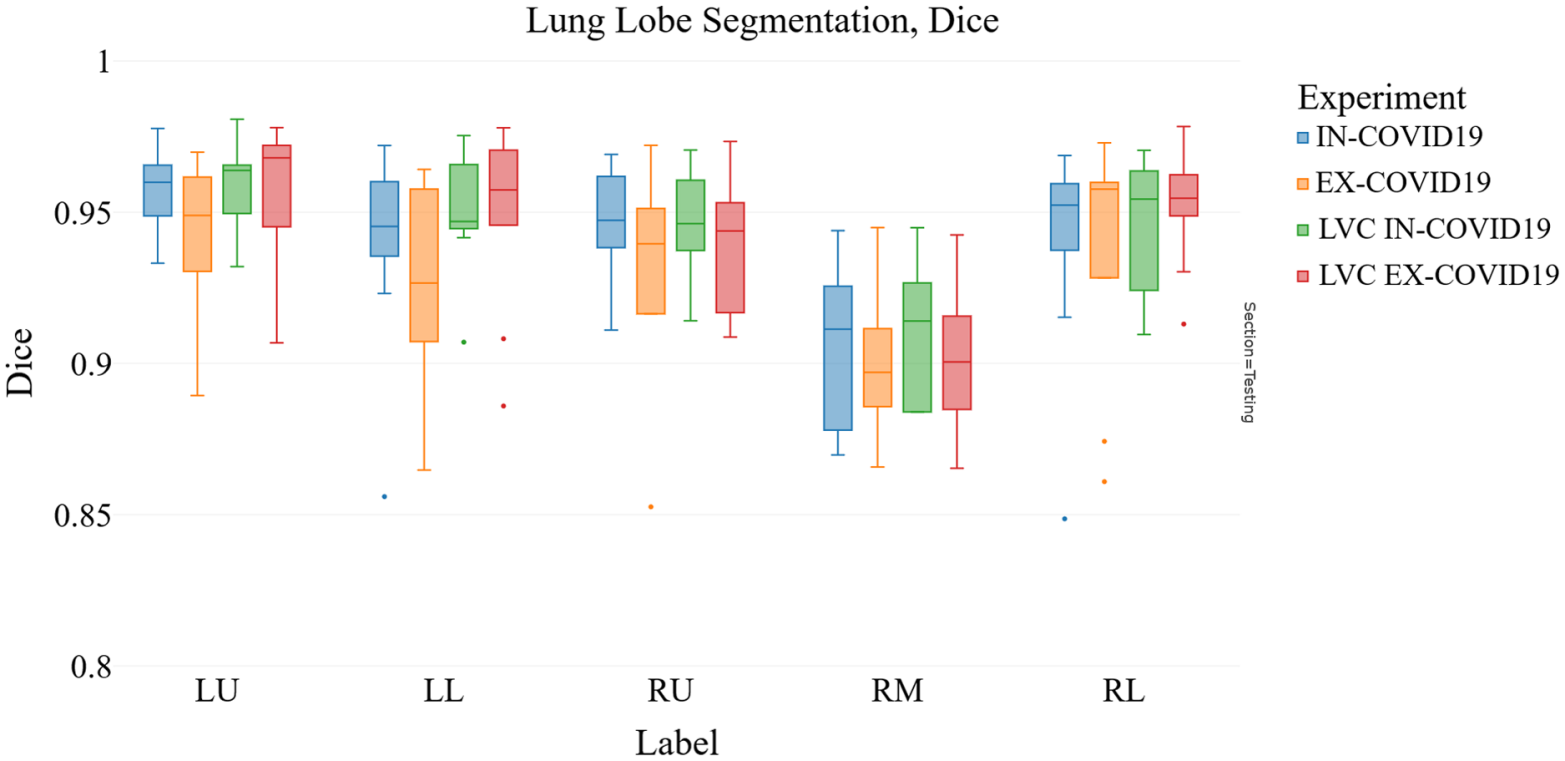
COVID-19 validation Dice score: comparison between two models trained in the expiration phase and validated with COVID-19 cases (LVX-EX-COVID19 and EX-COVID19), and two models trained in the inspiration phase and validated with COVID-19 cases (LVX-IN-COVID19 and IN-COVID19).

**Fig. 13:**
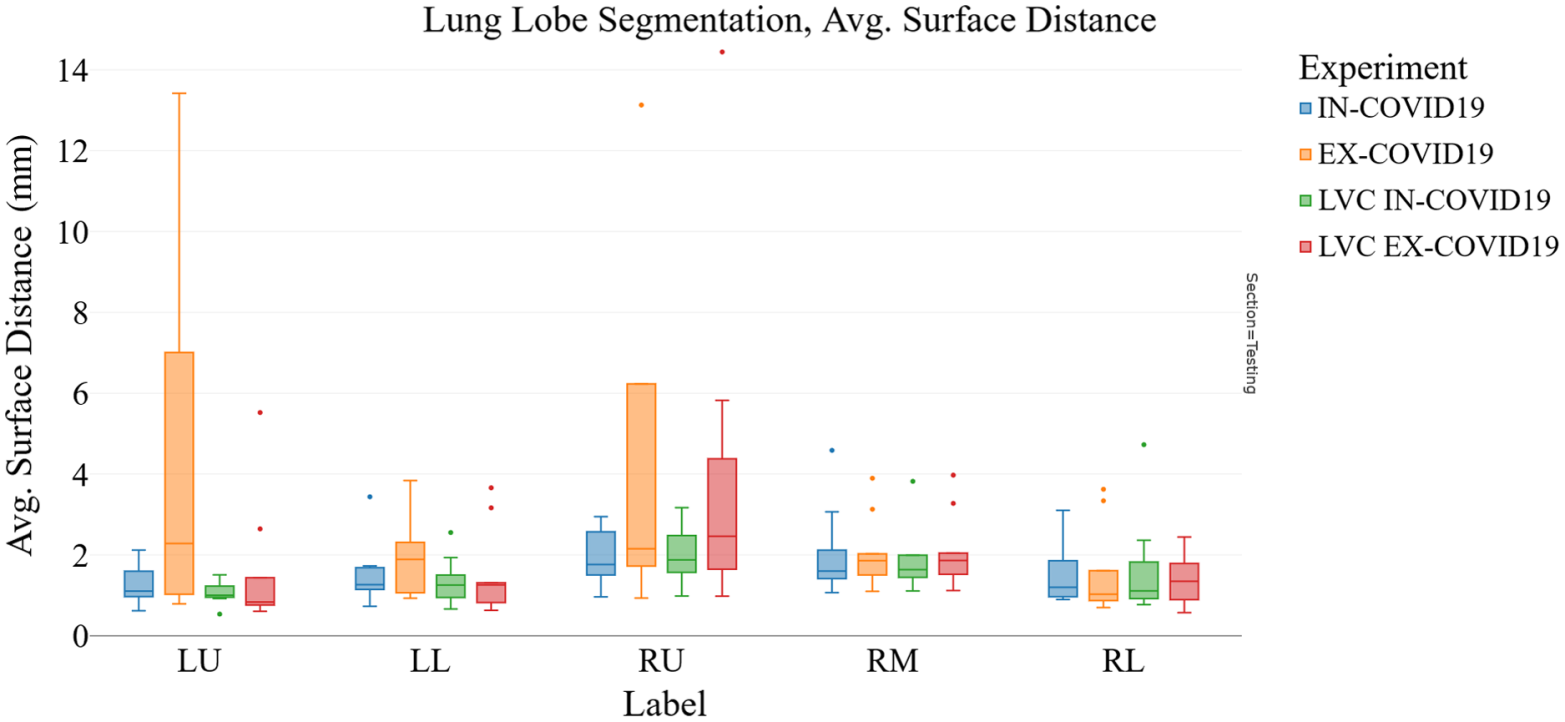
COVID-19 validation ASD: comparison between two models trained in the expiration phase (LVX-EX-COVID19 and EX-COVID19) and validated with COVID-19 cases, and two models trained in the inspiration phase (LVX-IN-COVID19 and IN-COVID19) and validated with COVID-19 cases. Note the performance improvement when including LVC in the EX-COVID19 models (baseline in orange, LVC model in red).

As illustrated in Fig. 12, the average Dice score for the expiration model increases from 0.92 to 0.93 when including the LVC information. Similarly, the ASD score is also improved on the expiration model (decreasing from 3.1 to 2.1 mm, Fig. 13).

Notably, relevant improvements were observed in the Dice and ASD performance for the left upper (LU) and left lower (LL) lobes. The Dice score for the LU lobe increased from 0.943 to 0.958 and for the LL lobe from 0.927 to 0.950. Similarly, the ASD for the LU lobe decreased from 4.3 to 1.5 mm and for the LL lobe from 2.0 to 1.5 mm, upon integrating LVC information into the model.

### 3.5 Multitasking Loss

As described in Sec. 2.3.2, we conducted a series of additional experiments on the multitasking network architecture to determine the optimal loss weights for lung lobe segmentation and fissure loss. We performed three separate experiments with loss weight combinations of (0.5, 0.5), (0.7, 0.3), and (0.9, 0.1).

As reported in Table 10, we identified (0.7, 0.3) as the optimal weight configuration. This selection is justified by demonstrating that the (0.5, 0.5) configuration did not adequately emphasize lobe segmentation performance, resulting in suboptimal outcomes. Conversely, the (0.9, 0.1) configuration was found to be too similar to the single-decoder LVC experiment, where lobe fissure segmentation is excluded from the loss computation.

**Table 10:** Mean Dice scores for lung lobe segmentation with different loss weights ($L_{l}$ is the lobe loss weight and $L_{f}$ is the Fissure loss weight) in multitasking experiments, including the single-decoder LVC experiment (LVC-EX) for comparison.

$L_{l}$	$L_{f}$		LU	LL	RU	RM	RL
0.5	0.5		0.954	0.965	0.909	0.816	0.964
0.7	0.3	(Selected)	0.969	0.967	0.965	0.937	0.971
0.9	0.1		0.963	0.967	0.956	0.928	0.965
1.0	0.0	(LVC-EX)	0.965	0.963	0.960	0.932	0.968

### 3.6 Statistical Analysis

To further validate our findings, we conducted a paired t-test to assess the significance of LVC information within the segmentation context under examination. The null hypothesis asserts that there is no average difference in the metrics presented (Dice and ASD). The resulting p-values for various experiment comparisons are detailed in Tables 11 and 12. As shown in Table 11, small but significant improvements in the Dice score are observed when comparing the baseline expiration experiment (Baseline-EX) with the corresponding multitasking one (MT-EX). In the context of interphase validation, notable results are seen when comparing the Baseline EX-IN model with the multitasking EX-IN model, where the observed Dice improvement reaches up to 7.5% in the multitasking model (for the LL lobe). However, only some of these improvements were statistically significant. Similar observations are made when analyzing the statistical test for improvements in ASD, where the mean ASD decreases by up to 8.6 mm (in the LU lobe) when comparing the multitasking EX-IN with its corresponding baseline.

**Table 11:** Comparison of Dice scores across different experiments, with n representing the number of cases examined. The p-values from paired t-tests are presented, testing the null hypothesis (H0) that the mean Dice scores of the compared experiments are equal. This table reports the signed average difference per lobe, where a positive value indicates an improvement in the average Dice score.

Comparison	Label	$E_{1}-E_{2}$
LVC-EX versus baseline-EX (n=59)	LU	**0.004** ***
	LL	**0.003** ***
	RU	**0.004** ***
	RM	**0.003** ***
	RL	**0.003** ***
LVC-IN versus baseline-IN (n=59)	LU	**0.001** ***
	LL	**0.003** ***
	RU	0.002
	RM	−0.001
	RL	**0.002** ***
MT-EX versus baseline-EX (n=59)	LU	**0.004** ***
	LL	**0.004** ***
	RU	**0.005** *
	RM	**0.005** *
	RL	**0.003** ***
MT-EX-IN versus baseline-EX-IN (n=11)	LU	**0.063** *
	LL	**0.075** *
	RU	0.008
	RM	0.019
	RL	0.012
LVC-EX-COVID versus EX-COVID (n=10)	LU	0.015
	LL	0.023
	RU	0.006
	RM	−0.001
	RL	0.014

The three standard significance p-value levels are illustrated as follows:

* p-value <0.01

** p-value <0.001

*** p-value <0.05

**Table 12:** Comparison of ASD across different experiments, with n representing the number of cases examined. The p-values from paired t-tests are presented, testing the null hypothesis (H0) that the mean ASD of the compared experiments are equal. This table reports the signed average difference per lobe, where a negative value indicates an improvement in the mean ASD.

Comparison	Label	$E_{1}-E_{2}$
LVC-EX versus baseline-EX (n=59)	LU	**−0.105** *
	LL	**−0.074** ***
	RU	−0.104
	RM	−0.137
	RL	**−0.051** ***
LVC-IN versus baseline-IN (n=59)	LU	**−0.071** *
	LL	**−0.086** ***
	RU	−0.112
	RM	−0.07
	RL	−0.047
MT-EX versus baseline-EX (n=59)	LU	**−0.122** *
	LL	**−0.074** ***
	RU	**−0.0156** **
	RM	−0.169
	RL	**−0.055** *
MT-EX-IN versus baseline-EX-IN (n=11)	LU	−8.575
	LL	**−1.488** *
	RU	−2.065
	RM	−1.647
	RL	−0.491
LVC-EX-COVID versus EX-COVID (n=10)	LU	−2.808
	LL	−0.446
	RU	−1.475
	RM	0.026
	RL	−0.19

The three standard significance p-value levels are illustrated as follows:

* p-value <0.01

** p-value <0.001

*** p-value <0.05

## 4 Discussion

In this study, we introduced a DL approach to enhance the segmentation of lung lobes in chest CT scans acquired during the expiration phase. Our primary innovation involved integrating prior anatomical information into the DL model in the form of an LVC map.

To assess the impact of LVC on segmentation performance, we conducted a thorough analysis and compared the results from our DL models in both single-phase (intraphase) and interphase scenarios to evaluate generalization robustness. Additionally, we examined the model’s effectiveness in segmenting lung lobes in the presence of lung lesions using a COVID-19 dataset.

**Fig. 14:**
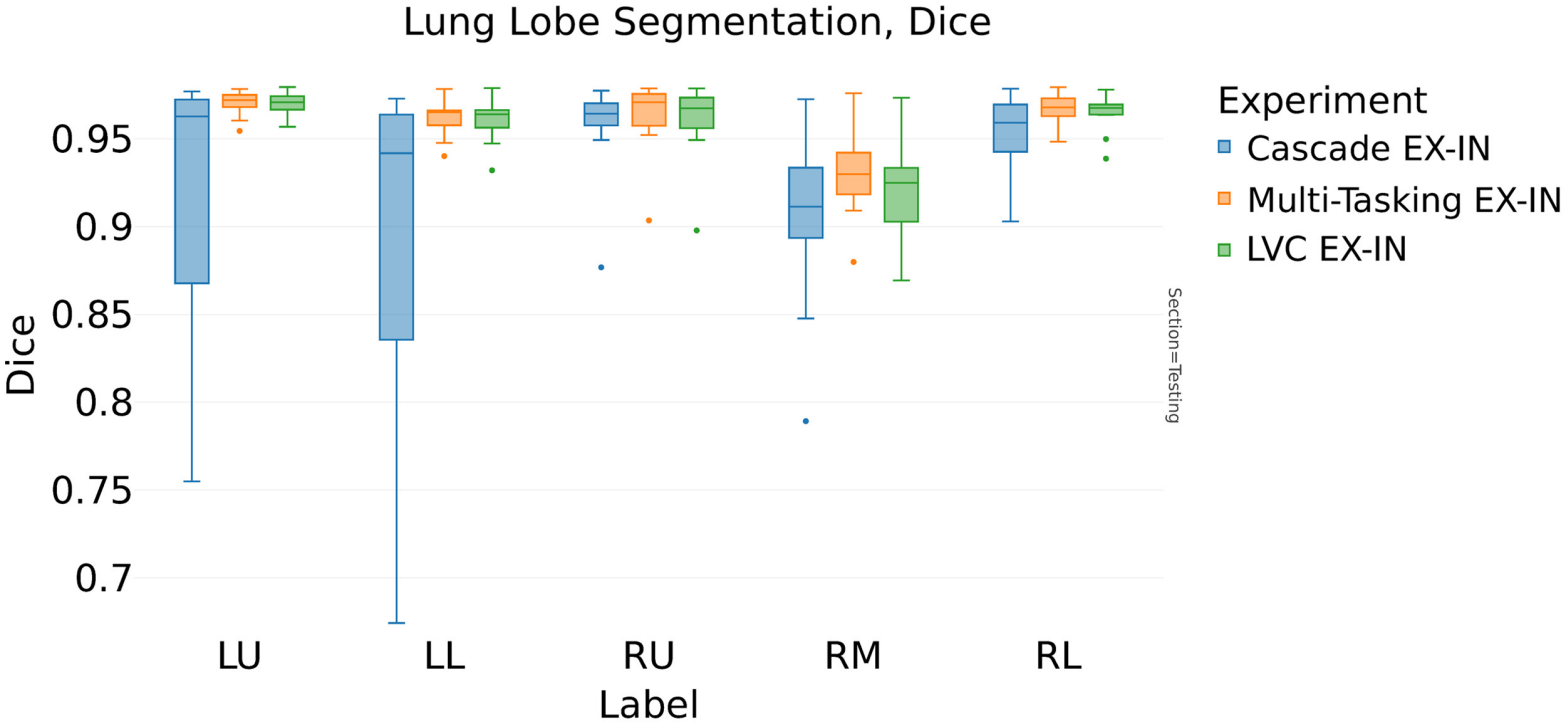
Interphase validation Dice score: LVC U-Net (LVC EX-IN), cascade, and multitasking model comparison trained in expiration phase and validated in inspiration phase (EX-IN).

Our findings, as discussed in Sec. 3, highlight the benefit of incorporating LVC as prior anatomical information into the DL model for improving lobar segmentation. Notably, we observed a positive influence of LVC information on local segmentation accuracy, particularly in the boundary regions of the lung lobes. This improvement is quantified through the analysis of ASD scores. In the case of single-phase models, the inclusion of LVC information led to a reduction in ASD, demonstrating its efficacy for both inspiration and expiration models (Table 5). Furthermore, our results indicate that adopting a multitasking U-Net architecture can further enhance segmentation performance in these boundary regions. Conversely, a two-step cascade approach did not significantly contribute to the segmentation results and, in some cases, even led to performance degradation, as shown in the interphase validation comparison (Figs. 11 and 14).

A plausible explanation for this discrepancy is error propagation from inaccurate fissure segmentation, which can result from variations in anatomical conformation between breathing phases. This issue is further intensified in the presence of pathological lung diseases, where incomplete fissures are common. As documented in the literature, segmentation performance significantly deteriorates under these conditions, supporting the hypothesis that errors in fissure segmentation contributed to the overall reduced performance in lobe segmentation.

We can draw similar conclusions regarding the generalization capabilities of the models. As depicted in Table 7, the incorporation of LVC information guides the model to achieve improved ASD performance, and the multitasking approach has a positive impact on the final segmentation results. The Dice score results, particularly for the expiration models (Table 6), support this notion in terms of generalizability.

Conversely, our models did not demonstrate a significant improvement in segmenting lung lobes in the presence of COVID-19 lesions. While the expiration model displayed slightly better performance with the inclusion of LVC information, the significance of these results cannot be confirmed based on Tables 12 and 11.

While optimal segmentation results can already be attained with traditional methods, our approach prioritizes local improvements in the segmentation of lobar boundary regions, as evident from the ASD results. This study’s potential applications lie in clinical scenarios where the accurate delineation of lung fissure regions, i.e., the boundary regions between lobes, is a primary requirement.

These findings will be valuable for future studies focusing on lobar boundary regions that demand enhanced segmentation accuracy. Additionally, adopting a multitasking approach can further improve segmentation results, enabling the simultaneous segmentation of lung lobes along with their corresponding fissures.

### 4.1 Study Limitations

Despite the promising outcomes observed in this study when incorporating prior anatomical knowledge into DL models for lung lobe segmentation in expiration chest CT scans, the same findings exhibit weaker support when applied to DL models trained with inspiration CT volumes.

Moreover, the modest enhancements seen in the average Dice score results can be attributed to the high performance already achieved with conventional DL-based segmentation methods, such as nnU-Net.^34^ These state-of-the-art methods yield satisfactory Dice scores, allowing limited room for further improvements.

Finally, it is important to exercise caution when interpreting the results related to COVID-19 lesions due to the study’s utilization of a limited number of COVID-19 cases (10). This limited sample size may not be fully representative of broader populations.

## 5 Conclusions

This study highlights the significant advantages of incorporating LVC information in lung lobe segmentation, particularly in the boundary regions between lobes. Additionally, our findings support the notion that the inclusion of LVC information enhances the generalization ability of the models. Notably, the models trained with expiration CT volumes exhibit improved generalization compared to those trained with inspiration volumes, as indicated by both Dice scores and ASD.

## Data Availability

The SCAPIS dataset, part of the study, is not publicly available. Access might be granted upon ethical approval. The COVID-19 dataset presented in this article is publicly available in DOI 10.5281/zenodo.3757476. The code described in this article can be freely accessed through GitHub, as a fork of the original nnU-Net v1 code: https://github.com/MIC-DKFZ/nnUNet.git.
